# Comparison of two distinct needle tip positions in pulsed radiofrequency for herpes zoster‐related pain

**DOI:** 10.1111/cns.14146

**Published:** 2023-03-07

**Authors:** Shao‐jun Li, Dan Feng

**Affiliations:** ^1^ Department of Pain Management Wuhan No. 1 Hospital Wuhan China

**Keywords:** DRG position, herpes zoster, needle tip position, pulsed radiofrequency

## Abstract

**Background:**

Herpes zoster (HZ)‐related pain, characterized by chronic and persistent pain with a dermatomal distribution, is a relatively common complication of HZ. Pulsed radiofrequency (PRF) can effectively relieve HZ‐related pain. There is no study on the effect of the needle tip position in patients with HZ for PRF treatment. This prospective study was conducted to compare two distinct needle tip positions in PRF for HZ‐related pain.

**Methods:**

Seventy‐one patients suffering from HZ‐related pain were enrolled in this study. According to the dorsal root ganglion (DRG) position and needle tip position, patients were randomly allocated to the IP group (group inside of the pedicle, *n* = 36) and OP group (group outside of the pedicle, *n* = 35). Quality of life and pain control were evaluated with the visual analog scale (VAS) and activities of daily living questionnaires (including 7 items: general activity, mood, walking ability, normal work, relations with other people, sleep, and enjoyment of life), which were administered before therapy and at intervals of 1, 7, 30, and 90 days after therapy.

**Results:**

Before therapy, the mean pain score was found to be 6.03 ± 0.45 in the IP group and 6.00 ± 0.65 in the OP group (*p* = 0.555). No significant differences were found when the two groups were compared at 1 and 7 days after therapy (*p* > 0.05). But, the pain score was significantly lower in the IP group at 30 days (1.78 ± 1.31 vs. 2.77 ± 1.31, *p* = 0.006) and 90 days of follow‐up (1.29 ± 1.19 vs. 2.15 ± 1.74, *p* = 0.041). Significant differences between the two groups in terms of general activity (2.39 ± 0.87 vs. 2.86 ± 0.77, *p* = 0.035), mood (1.97 ± 1.65 vs. 2.86 ± 1.50, *p* = 0.021), relations with other people (1.94 ± 0.92 vs. 2.51 ± 1.22, *p* = 0.037), sleep (1.64 ± 1.44 vs. 2.97 ± 1.44, *p* < 0.001), and enjoyment of life (1.58 ± 1.11 vs. 2.43 ± 1.33, *p* = 0.004) were detected after the 30‐day follow‐up. In addition, scores for the activities of daily living were significantly lower in the IP group than that in the OP group at 90 days after therapy (*p* < 0.05).

**Conclusion:**

The needle tip position had an influence on the PRF treatment in patients with HZ‐related pain. Positioning the needle tip in the area between the medial and lateral edges of adjacent pedicles offered good pain relief and improved quality of life in HZ patients.

## Graphical abstract

According to the dorsal root ganglion (DRG) position and needle tip position, patients were randomly allocated to the IP group (group inside of the pedicle) and OP group (group outside of the pedicle). This is the first study on two distinct needle tip positions in PRF for HZ‐related pain. In this study, it was shown that the patients in the IP group had significantly better pain relief and higher activities of daily living than that in the OP group.

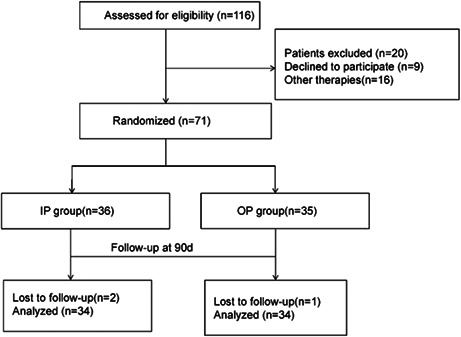

## 1 INTRODUCTION

Herpes zoster (HZ) is caused by the reactivation of latent varicella–zoster virus (VZV) and has an asymmetrical dermatomal rash and synchronous occurrence of skin lesions.^1^ HZ‐related pain, characterized by chronic and persistent pain with a dermatomal distribution, is a relatively common complication of HZ. Most of the patients with HZ experience symptoms of pain. If the pain persists at least 90 days after rash onset, it is defined as postherpetic neuralgia (PHN). The incidence of HZ and PHN is 25%–30% and 10%–20%, respectively.^2^, ^3^ It is well known that spontaneous ongoing pain could lead to anxiety, depression, and even physical and psychological dysfunction. The neuralgia could not only cause considerable suffering but also affect the quality of life, leading to a societal healthcare burden.^4^, ^5^ Therefore, pain control and improving quality of life in patients with HZ are considered the main goals in the evaluation of treatment options.

To date, pulsed radiofrequency (PRF) has been an effective option for pain control and the prevention of PHN. Kim et al.^6^ reported that PRF to the dorsal root ganglion (DRG) could provide better pain relief, particularly in patients who received PRF early after HZ onset. Similarly, Huang et al.^7^ showed that PRF treatment in the DRG was superior to that in the intercostal nerve, with improvements in visual analogue scale (VAS) and SF‐36 scores in PHN patients. It is well known that PRF is a technique that applies short pulses of RF signals. PRF occurs through RF generators which can produce transient pulsed RF current.^8^, ^9^ The RF generator uses alternatively repeated electrical stimulation, maintaining the temperature of the neural tissue below 42°C.^10^ In PRF, the short duration and resting phase between pulses can avoid apoptosis and necrosis of histocytes.^11^

Although PRF can offer pain relief, it faces some problems. Ding et al.^12^ found that the total effective rates of PRF in different stages (acute stage, subacute stage, and chronic stage) of HZ were 88%, 72%, and 52%, respectively. The authors concluded that the analgesic effect of PRF lasted longer in the acute phase but was lowest in the subacute and chronic phases. Luo et al.^13^ reported that PRF had a high short‐term recurrence rate in HZ patients. In addition, increasing evidence suggests that the voltage and the cycles were related to the therapeutic effect of PRF. High‐voltage and long‐duration PRF can reduce pain degree and improve the quality of life.^14^, ^15^ But, the voltage of PRF depends on the patient's tolerance. Not all patients were suitable for high‐voltage and long‐duration PRF. Moreover, high voltage may induce tissue damage.

It has been shown that PRF can reduce HZ‐related pain, but the poor efficacy of the treatment in some patients was quite confusing. At present, studies have demonstrated that needle tip position was significantly related to treatment effects.^16^, ^17^, ^18^ The DRG is the primary neuron for sensory transmission and the target structure in the PRF treatment. Positioning the needle tip to the optimal DRG targets is a key point for the success of the PRF treatment. In fact, placing the needle tip in the area between the medial and lateral edges of adjacent pedicles or outside of the pedicle can relieve pain in HZ patients. However, few studies have evaluated the effect of needle tip position on pain relief in HZ patients after PRF therapy. This study aimed to evaluate the profile of pain control and quality of life according to two different needle tip positions in PRF for HZ‐related pain.

## 2 METHODS

### 2.1 Patient selection

**FIGURE 1:**
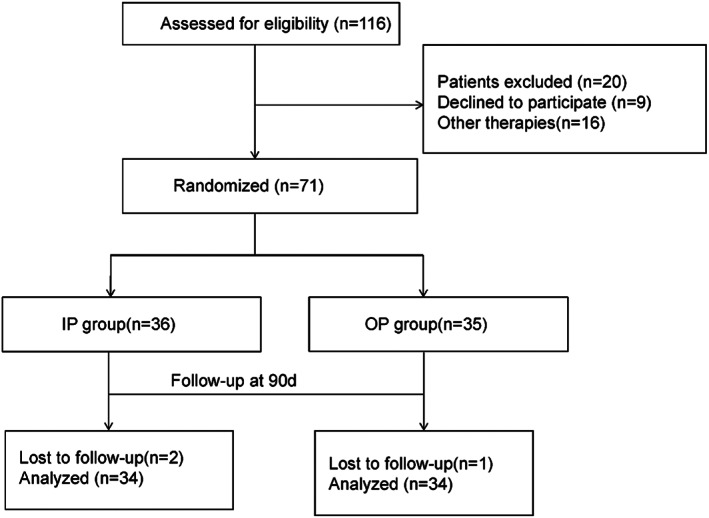
Flow diagram for the study.

**FIGURE 2:**
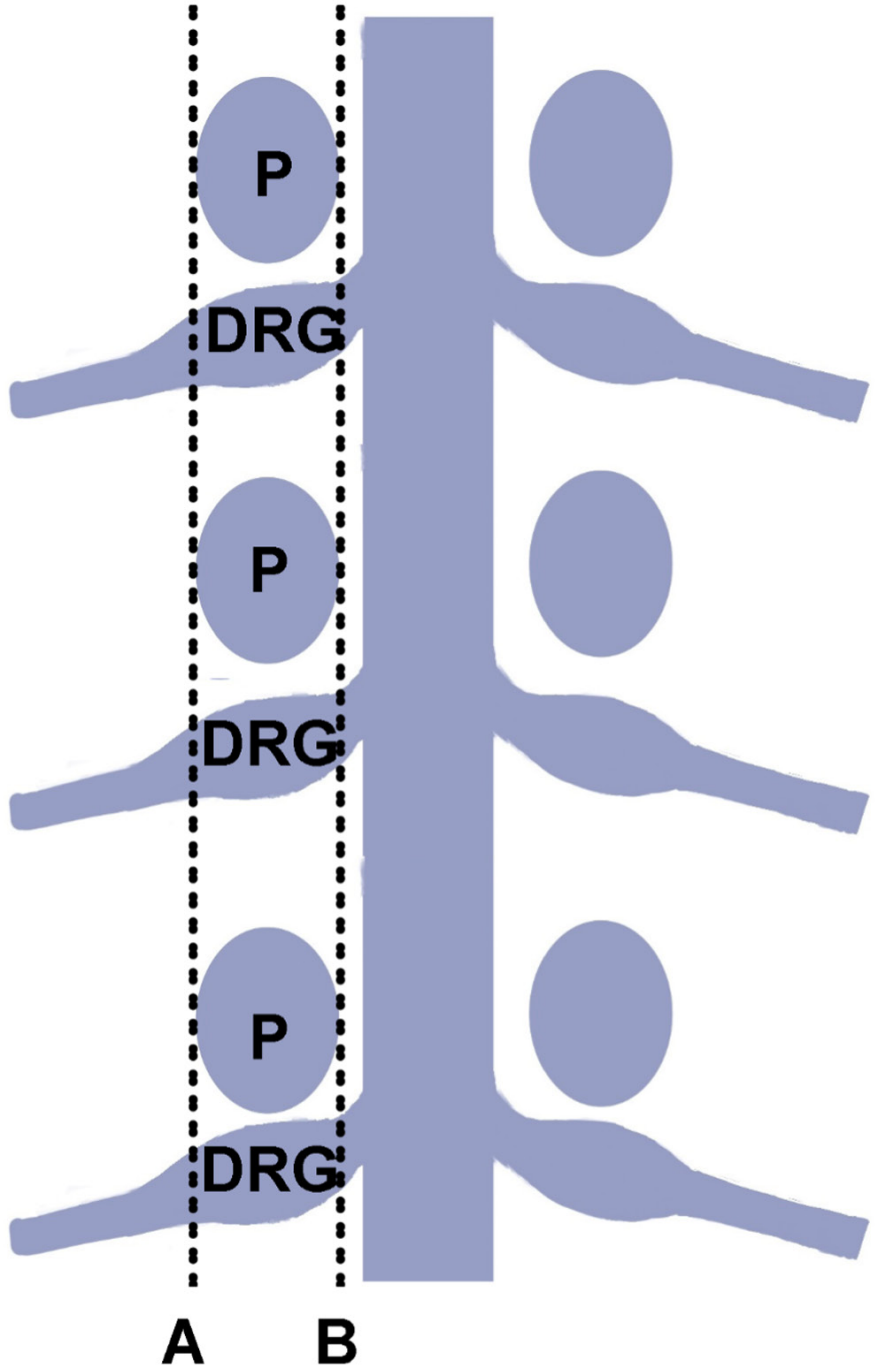
The DRG position and needle tip position. Lines A and B are lines connecting the adjacent medial and lateral margins of the pedicle. IP group: the final needle tip was located between lines A and B; OP group: the final needle tip was located lateral to line A. DRG, dorsal root ganglion; P, pedicle.

Between January 2018 and December 2019, 116 HZ patients who underwent PRF were enrolled at Wuhan No. 1 Hospital. All patients were randomized 1:1 in the study (Figure 1). According to tip position in PRF therapy, patients were randomly allocated to the IP group (group inside of the pedicle) and the OP group (group outside of the pedicle) by one physician using computer‐generated randomization sequences. Figure 2 shows the DRG position and needle tip position. Lines A and B are lines connecting the adjacent medial and lateral margins of the pedicle. According to the final anteroposterior view on fluoroscopic images, the IP group was defined as the final needle tip located between lines A and B. The OP group was defined as the final needle tip located lateral to line A. PRF therapy was carried out by one experienced doctor. Patients, researchers, and doctors were masked to group assignment.

The sample size was calculated by G power software (version 3.1), with a two‐sided 0.05 significance level and assuming an error of 5%. According to the reduced VAS scores and the effectiveness of PRF therapy (80% statistical power), a minimum of 35 patients per group were required.

The study was conducted in accordance with the principles of the Declaration of Helsinki and the guidelines of Wuhan No. 1 Hospital. All patients were asked to sign an informed consent document. The diagnosis of HZ was based on typical manifestations such as a vesicular, painful dermatomal rash.^1^ Inclusion criteria were as follows: age over 18 years, ability to speak Chinese, VAS score ≥4, barrier‐free communication, and thoracic or lumbar nerve root involvement. Disease‐related or patient‐related exclusion criteria were HZ with spinal tumor, thoracic or lumbar fracture, severe spinal stenosis, severely herniated disc, systemic infection, incomplete data in our center, and the failure of PRF therapy.

### 2.2 Measurements

The first step in treating HZ‐related pain was antiviral therapy and drug therapy (such as gabapentin, pregabalin, or analgesics). PRF therapy was considered for HZ patients who had not responded to medical therapy.

**FIGURE 3:**
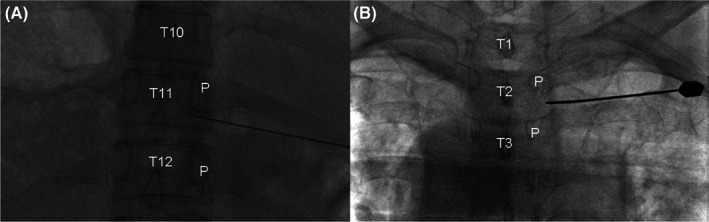
The final needle tip position in fluoroscopic images. (A) The needle was located between T11 and T12 pedicles. (B) The needle was located lateral to the T2 and T3 pedicles.

The PRF operation process was as follows. The patient was placed on the operating table in a prone position. The patient was connected to electrocardiogram monitoring, and low‐flow oxygen (2 L/min), ensuring the patient's vital signs were normal. The skin was disinfected and sterile covered. One‐percent lidocaine 3 mL was applied to the local anesthesia. Under digital subtraction angiography guidance, the needle was punctured into the target site (Figure 3). Once the final needle was close to the DRG position between lines A and B (IP group) or lateral to line A (OP group), a sensory stimulation test was conducted at 50 Hz, 0.5 v. The area evoked by the sensory stimuli was almost identical to the pain area in the herpes‐infected area. This criterion was judged to be a successful operation. Next, motor stimulation was tested at 2 Hz. PRF was performed at 42°C (electrode tip temperature), 2 Hz (pulse frequency), 20 ms (pulse width), 120 s (single duration), and three cycles. Finally, 4 mL of the liquid local anesthetic mixture (2% lidocaine 1 mL + dexamethasone 2 mL + 0.9% normal saline 1 mL) was injected into the DRG position.^19^

### 2.3 Questionnaire

The VAS and activities of daily living were used to evaluate pain intensity‐ and health‐related quality of life. VAS was measured on a scale from 0 to 10, where a line was about 10 cm. The two ends are the “0” end and the “10” end, where 0 indicates no pain, and 10 indicates the most severe pain. The activities of the daily living questionnaire were based on the Zoster Brief Pain Inventory questionnaire which was considered as a useful measure for quantifying herpes zoster pain, PHN, and impairment in daily living activities for clinical trials of herpes zoster prevention. The activities of the daily living questionnaire contain seven items including general activity, mood, walking ability, normal work, relations with other people, sleep, and enjoyment of life. Each question score referring to activities of daily living ranges from 0 to 10, where 0 indicates no interference and 10 indicates complete interference.^20^ In our study, the VAS and activities of daily living were assessed before therapy (baseline) and at intervals of 1, 7, 30, and 90 days after therapy.

### 2.4 Statistics

SPSS software (version 22.0 SPSS Inc., Chicago, IL) was used to perform the statistical analyses. Kolmogorov–Smirnov test was used to assess the normality of data distribution.

Demographic and clinical data for patients with HZ‐related pain were described using the mean ± SD and count (percentage) to represent continuous and categorical variables, respectively. A *t* test or chi‐square test was used to compare the data between the groups. The data of VAS and activities of daily living were assessed using the Mann–Whitney *U* test. Graphical representations were performed by Origin software (version 2022, Origin Lab, USA). *p* < 0.05 was considered statistically significant.

## 3 RESULTS

### 3.1 Patients characteristics

A total of 116 patients with HZ‐related pain were included in the study. Among them, 45 patients were excluded, and 71 patients who underwent PRF (IP group, *n* = 36; OP group, *n* = 35) were finally enrolled. Two patients in the IP group and one patient in the OP group were lost to follow‐up at 90 days after therapy. The characteristics of the included 71 patients are shown in Table 1. The mean age was 68.6 years in the IP group and 65.8 years in the OP group. The majority of the patients were female (58%). At inclusion, 76% (54/71) of patients had the persistence of pain for <3 months. There were 58 cases of HZ in the thoracic segment and 13 cases of HZ in the lumbar segment. Demographic and clinical characteristics data showed no statistically significant differences between the two groups (*p* > 0.05).

**TABLE 1:** Demographic and clinical characteristics data.

Variable	IP (*n* = 36)	OP (*n* = 35)	*p*
Age (SD)	68.6 ± 10.7	65.8 ± 11.2	0.281
Male/Female	17/19	13/22	0.390
Diabetes (%)	4 (11.1%)	5 (14.3%)	0.735
Hypertension (%)	15 (41.7%)	14 (40.0%)	0.886
Heart disease (%)	8 (22.2%)	11 (31.4%)	0.381
Malignancy (%)	1 (2.8%)	3 (8.6%)	0.293
Duration of pain (<3 months) (%)	26 (72.2%)	28 (80.0%)	0.443
Left/Right	12/24	15/20	0.409
HZ location (thoracic/lumbar)	28/8	30/5	0.387

*Note*: Data presented are means (SD) or numbers (%).

Abbreviations: HZ, herpes zoster; SD, standard deviation.

### 3.2 Pain and activities of daily living assessment

**FIGURE 4:**
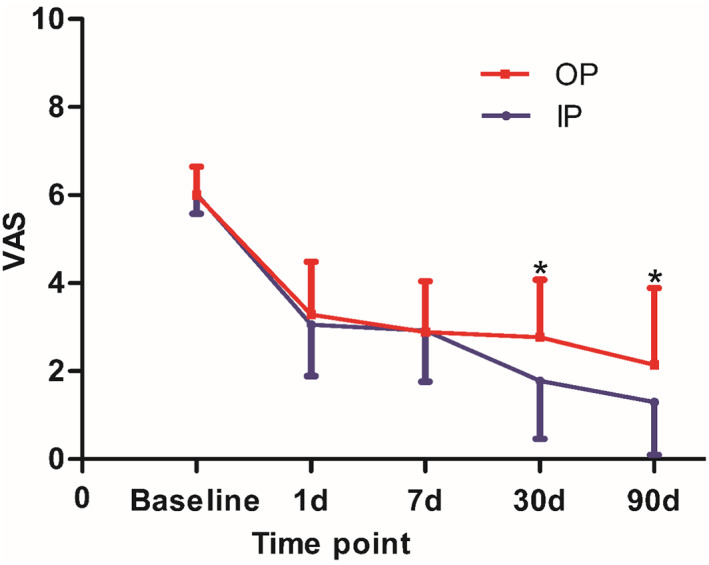
Results of the pain score over 90‐day follow‐up (*p < 0.05).

Before therapy, the mean pain score was found to be 6.03 ± 0.45 in the IP group and 6.00 ± 0.65 in the OP group (*p* = 0.555). No significant differences were found when the two groups were compared at 1 and 7 days after therapy (*p* > 0.05). But the pain score was significantly lower in the IP group at 30 days (1.78 ± 1.31 vs. 2.77 ± 1.31, *p* = 0.006) and 90‐day of follow‐up (1.29 ± 1.19 vs. 2.15 ± 1.74, p = 0.041) (Figure 4).

**FIGURE 5:**
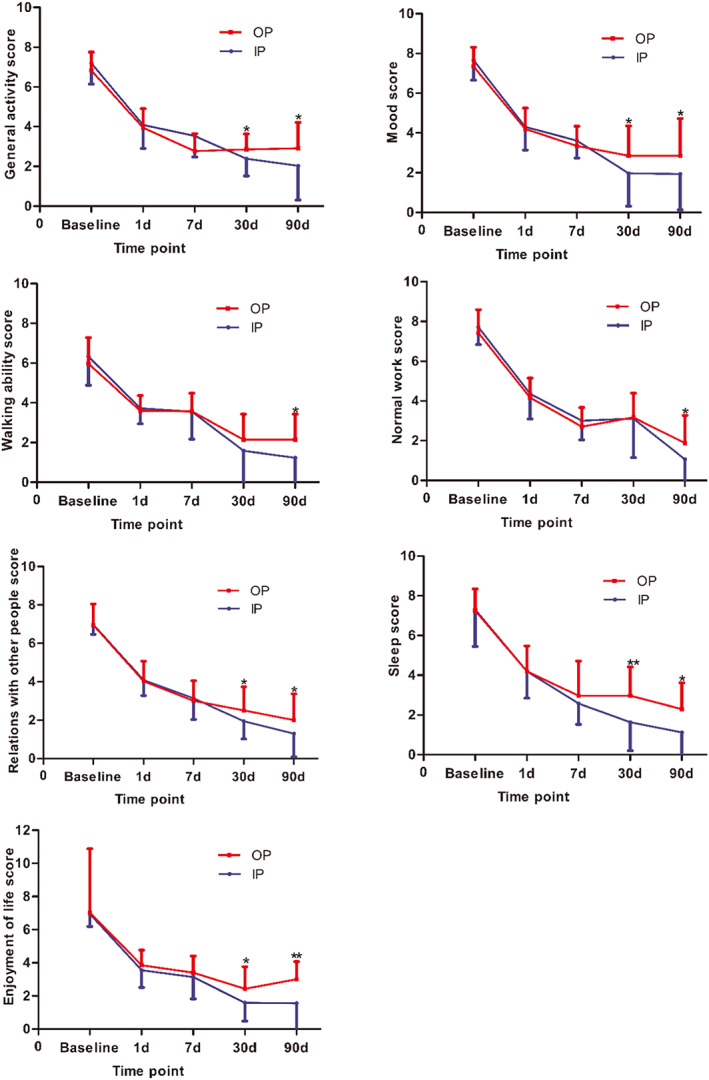
The score of HZ‐related pain on activities of daily living. Each question score referring to activities of daily living ranges from 0 to 10, where 0 indicates no interference and 10 indicates complete interference (*p < 0.05, **p < 0.001).

Figure 5 shows that both the two groups had equivalent outcomes with regard to activities of daily living before therapy, at 1 day, and 7 days after therapy (*p* > 0.05). Significant differences between the two groups in terms of general activity (2.39 ± 0.87 vs. 2.86 ± 0.77, *p* = 0.035), mood (1.97 ± 1.65 vs. 2.86 ± 1.50, *p* = 0.021), relations with other people (1.94 ± 0.92 vs. 2.51 ± 1.22, *p* = 0.037), sleep (1.64 ± 1.44 vs. 2.97 ± 1.44, *p* < 0.001), and enjoyment of life (1.58 ± 1.11 vs. 2.43 ± 1.33, *p* = 0.004) were detected after the 30‐day follow‐up. In addition, scores for the activities of daily living were significantly lower in the IP group than that in the OP group at 90 days after therapy (*p* < 0.05).

### 3.3 Adverse events

Numbness at the site of the skin puncture occurred in two patients in the IP group and one patient in the OP group. This symptom lasted for about 1 day and disappeared without any treatment. No other adverse events or neurological complications were found in the study.

## 4 DISCUSSION

This is the first prospective study on two distinct needle tip positions in PRF for HZ‐related pain. In this study, it was shown that the patients in the IP group had significantly better pain relief and higher activities of daily living than that in the OP group.

There is controversy in the treatment of HZ‐related pain with PRF. A systematic review in 2019 concluded that the evidence for most interventional procedures used to treat postherpetic neuralgia is Level 2, according to “The Oxford Levels of Evidence 2”.^21^ On the contrary, recent studies have shown that PRF effectively controls HZ‐related pain. Wang et al.^15^ showed that high‐voltage pulsed radiofrequency can relieve pain, improve sleep quality, reduce the doses of anticonvulsants and analgesics, and decrease the incidence of clinically meaningful PHN. Fei et al.^22^ confirmed that PRF combined with paravertebral injection of recombinant human interferon‐α2b was an effective treatment for acute stage herpes zoster neuralgia. Zhang et al.^23^ reported that PRF combined with intravenous lidocaine infusion can effectively relieve pain, reduce the number of analgesic drugs used, and improve sleep and quality of life. In fact, the DRG is a key structure in PRF treatment. Anatomically, the DRG is an enlargement of the dorsal root which is near the medial side of the intervertebral foramen. Kim et al.^6^ found that persistent abnormal electrical activity in the spinal cord through the DRG could lead to neuropathy in acute herpes zoster. The authors believed that the application of PRF to the DRG was a useful treatment for HZ‐related pain. Therefore, PRF to the DRG is an effective option for HZ‐related pain management interventions.

The mechanism of PRF for pain relief is unknown. Chen et al.^24^ injected the complete Freund's adjuvant (CFA) into the unilateral hind paw of rats to induce mechanical hyperalgesia in both the ipsilateral and contralateral hind paws. They concluded that the application of PRF close to the DRG was an effective treatment for CFA‐induced persistent mechanical hyperalgesia by attenuating spinal c‐Jun N‐terminal kinases (JNKs) activation in the spinal dorsal horn. Erdine et al.^25^ found that PRF may have a selectively greater effect on the smaller pain‐carrying fibers (C‐fiber and A‐delta fibers), and a lesser effect on the larger A‐beta neurons that mediate nonpain‐related sensations. In addition, Cho et al.^26^ reported that glutamate A1 (GluA1) and GluA2 subunits were upregulated in the radicular neuropathic pain model, and PRF stimulation induced the translocation of GluA1 and GluA2 subunits from the synaptosome into the cytosol. The authors concluded that PRF stimulation affected the synaptic plasticity corresponding to long‐term depression. Xu et al.^27^ showed that microglial brain‐derived neurotrophic factor (BDNF), phosphatidylinositol 3‐kinase (PI3K), and phosphorylated extracellular signal‐regulated kinase (p‐ERK) in the spinal cord were suppressed by PRF on the DRG to ease spared nerve injury‐induced neuropathic pain in rats. Currently, there are many different theory voices about the mechanism of PRF in pain relief. Therefore, convincing, well‐established theories require further research.

Clinically, although PRF can effectively relieve HZ‐related pain, there are still some patients with poor pain relief and the factors that influence the efficacy of PRF treatment are uncertain. At present, studies have found that needle tip position may affect the treatment efficacy. Irwin et al.^18^ have shown that needle tip position had an influence on the accuracy of selective nerve root block. Similarly, Lee et al.^17^ have reported that positioning the needle tip medial to the pedicle helped in the spread of thecontrast media into the epidural space during transforaminal epidural injection using Kambin's approach. On the contrary, Kim et al.^28^ reported the opposite. Kim and colleagues published the results of a retrospective observational study. Forty‐nine patients were classified into groups inside of the pedicle and groups outside of the pedicle based on needle tip position. The authors concluded that the analgesic efficacy of PRF treatment did not differ with the needle tip position at 4, 8, and 12 weeks. In our study, we compared two distinct needle tip positions (IP and OP group) in PRF for HZ‐related pain. At the end of the observation period, the IP group experienced a significant pain decrease and improved quality of life. The results showed that the pain score was significantly lower in the IP group at 30‐day (1.78 ± 1.31 vs. 2.77 ± 1.31, *p* = 0.006) and 90‐day follow‐up (1.29 ± 1.19 vs. 2.15 ± 1.74, *p* = 0.041). After the 30‐day follow‐up, scores for general activity, mood, relations with other people, sleep, and enjoyment of life were significantly lower in the IP group (*p* < 0.05). Unfortunately, our results differ compare with those reported by Kim and colleagues due to the following possible reasons. On one hand, it has been shown that the DRGs anatomy varies in the thoracic and lumbar spine. Shen et al.^29^ assessed the DRGs from L1 through L5 including the location, signal intensity, architecture, and dimensions. They reported that most DGRs were foraminal in location and a few of the L5 DGRs were located intraspinally. The sizes and dimensions of DRGs were different in the lumbar spine. On the other hand, the mechanism of HZ is different from that of chronic lumbar radicular pain. The underlying mechanism of HZ is that the reactivated virus can replicate and propagate along a nerve, resulting in an inflammatory immune response leading to cell necrosis and death within the nerve root and ganglion.^30^ However, chronic lumbar radicular pain is related to mechanical compression on DRGs. Whether PRF therapy has a different effect on DRG injury and mechanical stress is unknown. For mechanical stress on DRGs caused by chronic lumbar radicular pain, the analgesic efficacy of PRF treatment did not differ with the needle tip position. But for DRG injury caused by HZ, positioning the needle tip medial to the pedicle may provide better pain relief.

There were some limitations in the study. First, the main limitation of our study was that the sample size was small and the follow‐up period was short, which may affect the reliability of this study. Second, morphine equivalent consumption may not be quantified. We could not standardize pain therapies after discharge, resulting in a potential bias of follow‐up results. Third, the pathologic anatomy of the DRG may differ in the spine. Radiologically, there were three types of the location of the DRG (intraspinal, foraminal, and extraforaminal). In this study, we only considered foraminal and extraforaminal DRG because previous research found that the intraspinal types only occurred at the L5 level in the lumbar spine, and the incidence of the variation was 5.7%.^31^ In this study, PRF treatment of the DRG was applied to both the lumbar spine and the thoracic spine. The variants of the DRG may lead to biased findings. In addition, pain severity, character, and timing may vary in the course of HZ. The pain appears to be easier to control in the early stages of the disease and it can be difficult to relieve pain once it persists longer than 3 months. In our experience, some patients in the stage of postherpetic neuralgia did not respond to medical therapy, nerve block, PRF therapy, and spinal cord stimulators. They required analgesics and eventually became addicted. Therefore, the pain having many forms in different stages of HZ may have an impact on the effect of PRF.

In conclusion, the position of the needle tip had an influence on the PRF treatment in patients with HZ‐related pain. Positioning the needle tip in the area between the medial and lateral edges of adjacent pedicles offered good HZ‐related pain relief and improved quality of life.

## CONFLICT OF INTEREST STATEMENT

The authors have no funding and conflicts of interest to disclose.

## Data Availability

Detailed data are available from the corresponding author upon reasonable request.
